# Transoral robotic surgery and neck dissection for hypopharyngeal cancer: long-term prognostic factors and survival outcomes

**DOI:** 10.1007/s11701-026-03171-5

**Published:** 2026-02-04

**Authors:** Wen-Chun Lin, Man-Wei Hua, Tian-Yun Lin, Jing-Jie Wang, Shih-An Liu, Kai-Li Liang, Eugene N. Myers, Chen-Chi Wang

**Affiliations:** 1 Department of Otorhinolaryngology Head and Neck Surgery, Taichung Veterans General Hospital, Taichung, Taiwan; 2 School of Medicine, College of Medicine, National Yang Ming Chiao Tung University, Hsinchu, Taiwan; 3 Institute of Clinical Medicine, National Yang Ming Chiao Tung University, Hsinchu, Taiwan; 4 Department of Post-Baccalaureate Medicine, College of Medicine, National Chung Hsing University, Taichung, Taiwan; 5 Department of Otolaryngology, University of Pittsburgh School of Medicine, Pittsburgh, PA USA

## Abstract

Transoral robotic surgery (TORS) with neck dissection has emerged as an organ-preserving treatment for hypopharyngeal squamous cell carcinoma (HPSCC), but long-term evidence remains limited. This retrospective study evaluated oncologic outcomes, prognostic factors, mortality patterns, and organ preservation in patients with T1–T3 HPSCC. From October 2010 to August 2023, 48 patients without prior upper aerodigestive tract malignancy or irradiation underwent TORS with neck dissection, with or without cisplatin-based neoadjuvant chemotherapy and adjuvant chemoradiation. Tumor stages included T1 (37.5%), T2 (45.8%), and T3 (16.7%), and en bloc resection was achieved in all cases. Pathologic analysis revealed lymph node metastasis in 46.8% and extranodal extension in 17%. Radiotherapy to the primary hypopharynx was omitted in 60% of patients; when administered, the mean dose was approximately 60 Gy. After a mean follow-up of 5.9 ± 3.5 years, 5- and 10-year recurrence-free survival rates were both 69%, while disease-specific survival rates were 77%. Overall survival at 5 and 10 years was 77% and 59%, respectively. Survival and recurrence were significantly associated with extranodal extension in multivariable analysis (*p* < 0.05). Of 15 deaths, distant metastases (46.7%) and second primary malignancies (33.3%) were predominant, whereas local recurrence accounted for only 13.3%. TORS with neck dissection provides durable disease control and excellent organ preservation for T1–T3 HPSCC, allowing radiotherapy omission in selected patients, although distant metastases and secondary cancers remain major causes of late mortality.

## Introduction

Cancer of the hypopharynx is among the most aggressive subtypes of squamous cell cancer of the head and neck(HNSCC), typically presents at an advanced stage and carries a poor prognosis, with a reported 5-year overall survival of approximately 30%–35% [1]. Prognosis is strongly stage-dependent: 5-year survival is approximately 60% for early cancers (T1–T2) but declines to < 25% for patients with more advanced cancers (T3–T4) or cervical lymph node metastasis [2, 3].

Traditional open surgical approaches are associated with substantial morbidity and functional impairment, particularly affecting swallowing and speech. In 1996, JL Lefebvre et al. reported a phase III trial of neoadjuvant chemotherapy followed by radiotherapy for laryngeal preservation in pyriform sinus cancer [4]. Chemoradiation subsequently became a common strategy for treating hypopharyngeal cancer. However, because most participants (74%) in that trial had clinical T3 disease, whether non-surgical organ-preservation therapy is the optimal treatment for early T1–T2 hypopharyngeal cancer requires further investigation. Studies [5, 6]. have indicated that the overall rate of cervical lymph-node metastasis among patients with hypopharyngeal cancer is as high as 59%; even in patients without clinically palpable cervical lymphadenopathy (cN0), the risk of occult nodal disease is approximately 36%–56%. A patient with T1–T2 hypopharyngeal cancer may therefore be assigned overall stage III or IV if neck metastasis is identified. In addition, a recent meta-analysis [7] demonstrated that primary surgery yielded better overall survival (OS) and disease-free survival (DFS) than concurrent chemoradiation (CCRT). Accordingly, a transoral approach combined with neck dissection may minimize surgical trauma and help guide adjuvant therapy for T1, T2, and selected T3 hypopharyngeal cancers.

Transoral robotic surgery (TORS) has been widely adopted for oropharyngeal cancer as a minimally invasive transoral technique, and Constantino et al. [8] reported that primary transoral surgery offers better survival outcomes for elderly patients with HPV-related oropharyngeal cancer compared with primary radiotherapy. Similarly, some reports [9–11] have shown encouraging results using TORS for hypopharyngeal cancers in terms of oncologic control and postoperative functional recovery. Our preliminary study also demonstrated promising outcomes [12, 13].

However, hypopharyngeal cancer is rare and accounts for less than 5% of all head and neck malignancies [14]. Evidence regarding TORS remains limited and is largely confined to short-term follow-up. This study therefore evaluates long-term oncologic outcomes and factors associated with survival.

## Methods

This retrospective cohort study was conducted in the Department of Otorhinolaryngology–Head and Neck Surgery at Taichung Veterans General Hospital (TCVGH), the only public tertiary referral medical center in central Taiwan and the first Head & Neck Robotic Surgery Observation Site approved by Intuitive Surgical (Sunnyvale, CA, USA). After institutional review board approval (IRB TCVGH No: CE25414A), the study was performed from 2010 to 2025 in accordance with the Strengthening the Reporting of Observational Studies in Epidemiology (STROBE) Statement [15].

### Study cohort

From October 2010 to August 2023, 211 consecutive patients with newly diagnosed or recurrent upper aerodigestive tract malignancies underwent TORS at TCVGH. After excluding oropharyngeal cancer, laryngeal cancer, and recurrent hypopharyngeal cancer, forty-eight patients with hypopharyngeal cancer classified as T1–T3 (AJCC 8th edition) and without any history of other head and neck malignancy or prior upper aerodigestive tract irradiation received TORS with simultaneous neck dissection, with or without neoadjuvant chemotherapy. All patients received pre-operative computed tomography which was covered by National Health Insurance to radiologically stage primary and neck disease. Demographics; clinical and pathologic TNM staging per the AJCC Cancer Staging System, 8th Edition; perioperative details; adjuvant therapy; and follow-up data were retrieved from the institutional database to evaluate long-term outcomes of this management strategy.

### Management

All patients with T1–T3 upper aerodigestive tract cancer underwent TORS-assisted surgery using the da Vinci S, Si or Xi system (Intuitive Surgical, Sunnyvale, CA, USA) after informed consent was obtained. Cisplatin-based neoadjuvant chemotherapy (NACT) was administered to selected patients with T3 cancer and to some patients with early T1–T2 tumors who could not undergo TORS promptly after diagnosis. All TORS procedures entailed en-bloc resection of the hypopharyngeal cancer and were performed under general anesthesia by the corresponding author with assistance from the first author, most often without tracheostomy, as described in our published paper [12, 13]. Simultaneous ipsilateral neck dissection was recommended in most cases; but bilateral neck dissections were performed when bilateral metastatic disease was suspected clinically. Only one clinically N0 patient declined neck dissection and was managed under watchful observation. Resection proceeded until all intraoperative frozen-section margins were negative. On permanent pathology, margins of the main specimen were dichotomized as “margin free” or “margin positive.” Adjuvant treatment was determined according to pathologic findings, with multidisciplinary tumor board consensus for each case. In our hospital, postoperative radiotherapy ± chemotherapy is recommended for patients with T3 cancer, N2–N3 nodal disease, perineural invasion, lymphovascular invasion, extracapsular extension, or positive margins; the final plan was made through shared decision-making with the patient.

After treatment, patients were followed regularly with flexible laryngoscopy; chest radiography; computed tomography (CT) and/or magnetic resonance imaging (MRI); abdominal ultrasonography; whole-body bone scan; and positron emission tomography (PET/CT) to detect local recurrence in the hypopharynx, regional cervical recurrence, or distant metastasis.

### Statistical analysis

With long-term follow-up, Kaplan–Meier methods were used to estimate overall survival (OS), disease-specific survival (DSS), and recurrence-free survival (RFS). Variables potentially associated with survival—including sex, tumor site, clinical stage, pathologic stage, margin status, NACT, adjuvant radiotherapy, local recurrence, regional recurrence, and distant metastasis—were summarized and analyzed. Categorical variables were compared using the χ² test, Fisher’s exact test, or the Fisher–Freeman–Halton exact test. Ordinal (ranked) or continuous variables were analyzed using the Mann–Whitney U test. A two-sided p value < 0.05 was considered statistically significant. A Cox proportional hazards regression model was also used for multivariate analysis. Additional logistic regression was performed as sensitivity analyses.

## Results

Successful TORS was achieved in all 48 patients (47 men, 1 woman). Forty-three patients (89.6%) underwent TORS without temporary tracheostomy using oro-endotracheal intubation. Perioperative data are summarized in Table 1. Ages ranged from 38 to 84 years (mean [SD], 57.11 [9.99]). A history of smoking cigarettes, alcohol use, and betel-nut chewing was present in 83.3%, 77.1%, and 47.9% of patients, respectively. Two patients had prior malignancies outside the head and neck: one had rectal cancer resected 6 years before TORS, and another had lung adenocarcinoma and basaloid squamous cell carcinoma of the esophagus 3 years before TORS. Primary sites were the pyriform sinus in 40 patients (83.3%), posterior pharyngeal wall in 7 (14.6%), and pyriform sinus with partial postcricoid mucosa in 1 (2%). Clinically, 18 (37.5%) had T1, 22 (45.8%) had T2, and 8 (16.7%) had T3 disease, with nodal status ranging from N0 (54.2%) to N3 (16.7%). Overall, 24 patients (50%) had stage I–II disease and 24 (50%) had stage III–IV disease.

**Table 1:** Perioperative data of 48 patients who had cancer of the hypopharynx treated with TORS

	Total (*n *= 48)		Total (*n *= 48)
Age, median (IQR)	54.82 (52.34 - 63.66)	Sites, n (%)	
Male, n (%)	47 (97.92%)	pyriform sinus	40 (83.33%)
Smoke/Alchoc/Betal, n (%)	43 (89.58%)	post pharyngeal wall	7 (14.58%)
Smoke, n (%)	40 (83.33%)	pyriform sinus+post cricoid mucosa	1 (2.08%)
Alcohol, n (%)	37 (77.08%)	Laterality, n (%)	
Betal, n (%)	23 (47.92%)	Right	24 (50%)
1^ST^ ca. in life, n (%)	46 (95.83%)	Left	22 (45.83%)
Hx of other ca., n (%)	2 (4.17%)	Bilateral	2 (4.17%)
Diagnosis site, n (%)		Pathology T stage, n (%)	
In hospital	36 (75%)	T0	1 (2.44%)
Outside hosptial	12 (25%)	T1	26 (63.41%)
Clinical T stage, n (%)		T2	11 (26.83%)
T1	18 (37.5%)	T3	3 (7.32%)
T2	22 (45.83%)	Pathology N stage, n (%)	
T3	8 (16.67%)	N0	25 (53.19%)
Clinical N stage, n (%)		N1	11 (23.4%)
N0	26 (54.17%)	N2	5 (10.64%)
N1	10 (20.83%)	N3	6 (12.77%)
N2	10 (20.83%)	Pathology overall stage, n (%)	
N3	2 (4.17%)	I	15 (36.59%)
Clinical overall stage, n (%)		II	4 (9.76%)
I	12 (25%)	III	11 (26.83%)
II	12 (25%)	IVA	5 (12.2%)
III	12 (25%)	IVB	6 (14.63%)
IVA	10 (20.83%)	Pathology overall stage, n (%)	
IVB	2 (4.17%)	I	15 (36.59%)
Clinical overall stage, n (%)		II	4 (9.76%)
I	12 (25%)	III	11 (26.83%)
II	12 (25%)	IV	11 (26.83%)
III	12 (25%)	Pathologic differentiation, n (%)	
IV	12 (25%)	wd	1 (2.5%)
Neoadjuvant CT, n (%)	16 (33.33%)	md	17 (42.5%)
Temporary tracheostomy, n (%)	5 (10.42%)	md to pd	14 (35%)
Neck dissection, n (%)		pd	8 (20%)
ipsilateral	45 (93.75%)	Hp Permanent specimen margin, n (%)	
bilateral	2 (4.17%)	Negative	34 (72.34%)
Adjuvant RT, n (%)		Positive	13 (27.66%)
no	23 (47.92%)	Margin distance (mm), median (IQR)	1 (1 - 2)
neck	6 (12.5%)	Lymphovascular invasion, n (%)	10 (20.83%)
hypopharynx	2 (4.17%)	Node ENE, n (%)	8 (17.02%)
neck+hypopharynx	17 (35.42%)	Follow-up time, years (TORS date), median (IQR)	6.22 (2.33 - 8.65)
RT dose neck cGy, n (%)	23 (47.92%)	Death, any cause, n (%)	15 (31.25%)
RT dose neck cGy, median QR)	6000 (5400 - 6600)	Death, hypopharyngeal ca., n (%)	10 (23.26%)
RT dose Hp cGy, n (%)	19 (40.43%)	Recurrence (local, regional, distant), n (%)	14 (29.17%)
RT dose Hp cGy, median (IQR)	6000 (5940 - 6600)	Organ preserved, n (%)	47 (97.92%)
Adjuvant CT, n (%)	12 (25%)	Nasogastric tube dependency, n (%)	6 (12.5%)
Frozen number, median (IQR)	5 (4 - 5)	2nd primary ca., n (%)	23 (47.92%)

### TORS and neck dissection with/without neoadjuvant chemotherapy

Sixteen patients (33.3%) received neoadjuvant chemotherapy (NACT). The treatment duration of NACT ranged from 1 to 3 cycles, and the time interval between the last cycle of chemotherapy and the TORS surgery ranged from 6 to 24 days with a median(IQR) of 13(8–20) days. All primary tumors responded to NACT. Seven patients (44%) achieved a complete response (CR), confirmed by postoperative pathology showing pT0 status. The remaining nine patients (56%) demonstrated a partial response (PR), defined as at least a 30% reduction in the sum of diameters of target lesions.

To ensure negative margins, 2–10 frozen sections were submitted intraoperatively (median [IQR], 5 [4–5]). Forty-five patients (93.8%) underwent an ipsilateral neck dissection and 2 (4.2%) had bilateral neck dissections; 1 clinically N0 patient declined neck dissection (PET-CT negative) and was analyzed as pathologic N0. Negative margins on the en-bloc specimen were obtained in 34 patients (72.3%) with a median (IQR) margin distance of 1 (1–2) mm. Nineteen patients (46.3%) had overall pathologic stage I–II disease and 22 (53.7%) had stage III–IV disease. Lymph-node metastasis was confirmed in 22 patients (46.8%), and extranodal extension (ENE) in 8 (17%); thus, 36.3% (8/22) of node-positive cases had ENE. Lymphovascular invasion was present in 10 patients (20.83%). Concordance between clinical and pathologic N category was limited: only 29 patients (60.4%) were concordant (Table 2).

**Table 2:** Confusion matrix comparing the clinical and pathologic nodal metastasis staging

	Pathologic *N* (-)	Pathologic *N* (+)
Clinical N (-)	16	9
Clinical N (+)	10	13

### Adjuvant radiotherapy and chemotherapy

After surgery, 17 patients (35.4%) received irradiation to both neck and hypopharynx; 2 (4.2%) received irradiation to the hypopharynx only; and 6 (12.5%) received irradiation to the neck only. Nearly half of the cohort (23 patients, 47.9%) did not receive postoperative irradiation. Radiation doses ranged from 5000 to 7000 cGy, with median (IQR) 6000 cGy (5400–6600 cGy) to the neck and 6000 cGy (5940–6600 cGy) to the hypopharynx. Postoperative adjuvant chemotherapy was administered to 12 patients (25%).

### Survival, recurrence and associated factors

All patients were followed regularly after TORS for a median (IQR) of 6.22 (2.33–8.65) years. Survival status was additionally monitored by a dedicated nurse; for deaths occurring at home, time and cause were confirmed with family members. Fifteen patients (31.25%) died during follow-up. Table 3 compares survivors (*n* = 33) with decedents (*n* = 15): the mortality group had significantly higher pathologic N category, higher overall pathologic stage, and more frequent ENE. The mean radiation dose to the neck was higher in the mortality group (6600 cGy) than in survivors (5670 cGy). Adjuvant chemotherapy was also more common in the mortality group (46.7% vs. 15.2%). Table 4 compares the no-disease-recurrence group (*n* = 34) with recurrence group (*n* = 14), similarly the disease recurrence group also had significantly higher pathologic N category, higher overall pathologic stage, and more frequent ENE. However, after multivariate analysis (Tables 5 and 6), ENE seemed to be the most important factor associated with a significant higher hazard ratio in mortality and recurrence. Therefore, the higher radiation dose or adjuvant chemotherapy percentage in the mortality group more likely represents an indication of treatment for disease severity.

**Table 3:** Comparison of data between survival group and mortality group

Death	No (*n *= 33)	Yes (*n *= 15)	*p* value
Age, median (IQR)	56.31 (52.92 - 66.74)	52.81 (43.98 - 59.98)	0.087
Male, n (%)	32 (96.97%)	15 (100%)	1
Smoke/Alchoc/Betal, n (%)	29 (87.88%)	14 (93.33%)	1
Smoke, n (%)	27 (81.82%)	13 (86.67%)	1
Alcohol, n (%)	23 (69.7%)	14 (93.33%)	0.136
Betal, n (%)	13 (39.39%)	10 (66.67%)	0.08
1ST ca. in life, n (%)	33 (100%)	13 (86.67%)	0.093
Hx of other ca., n (%)	0 (0%)	2 (13.33%)	0.093
Diagnosis site, n (%)			1
In hospital	25 (75.76%)	11 (73.33%)	
Outside hosptial	8 (24.24%)	4 (26.67%)	
Clinical T stage, n (%)			0.369
T1	14 (42.42%)	4 (26.67%)	
T2	15 (45.45%)	7 (46.67%)	
T3	4 (12.12%)	4 (26.67%)	
Clinical N stage, n (%)			0.202
N0	21 (63.64%)	5 (33.33%)	
N1	5 (15.15%)	5 (33.33%)	
N2	6 (18.18%)	4 (26.67%)	
N3	1 (3.03%)	1 (6.67%)	
Clinical overall stage, n (%)			0.234
I	11 (33.33%)	1 (6.67%)	
II	9 (27.27%)	3 (20%)	
III	6 (18.18%)	6 (40%)	
IVA	6 (18.18%)	4 (26.67%)	
IVB	1 (3.03%)	1 (6.67%)	
Clinical overall stage, n (%)			0.138
I	11 (33.33%)	1 (6.67%)	
II	9 (27.27%)	3 (20%)	
III	6 (18.18%)	6 (40%)	
IV	7 (21.21%)	5 (33.33%)	
Pathology T stage, n (%)			0.486
T0	0 (0%)	1 (7.69%)	
T1	19 (67.86%)	7 (53.85%)	
T2	7 (25%)	4 (30.77%)	
T3	2 (7.14%)	1 (7.69%)	
Pathology N stage, n (%)			0.003**
N0	17 (51.52%)	8 (57.14%)	
N1	11 (33.33%)	0 (0%)	
N2	4 (12.12%)	1 (7.14%)	
N3	1 (3.03%)	5 (35.71%)	
Pathology overall stage, n (%)			0.004**
I	10 (35.71%)	5 (38.46%)	
II	2 (7.14%)	2 (15.38%)	
III	11 (39.29%)	0 (0%)	
IVA	4 (14.29%)	1 (7.69%)	
IVB	1 (3.57%)	5 (38.46%)	
Pathology overall stage, n (%)			0.018*
I	10 (35.71%)	5 (38.46%)	
II	2 (7.14%)	2 (15.38%)	
III	11 (39.29%)	0 (0%)	
IV	5 (17.86%)	6 (46.15%)	
Sites, n (%)			0.769
pyriform sinus	28 (84.85%)	12 (80%)	
post pharyngeal wall	4 (12.12%)	3 (20%)	
pyriform sinus+post cricoid mucosa	1 (3.03%)	0 (0%)	
Laterality, n (%)			0.146
R	17 (51.52%)	7 (46.67%)	
L	16 (48.48%)	6 (40%)	
RL	0 (0%)	2 (13.33%)	
Induction CT, n (%)	9 (27.27%)	7 (46.67%)	0.186
Tracheostomy, n (%)	3 (9.09%)	2 (13.33%)	0.642
Neck dissection, n (%)			0.379
ipsilateral	31 (93.94%)	14 (93.33%)	
bilateral	2 (6.06%)	0 (0%)	
Adjuvant RT, n (%)			0.165
no	18 (54.55%)	5 (33.33%)	
neck	2 (6.06%)	4 (26.67%)	
hp	1 (3.03%)	1 (6.67%)	
neck+hp	12 (36.36%)	5 (33.33%)	
RT dose neck cGy, n (%)	14 (42.42%)	9 (60%)	0.259
RT dose neck cGy, median (IQR)	5670 (5030 - 6150)	6600 (6000 - 6800)	0.010*
RT dose Hp cGy, n (%)	13 (40.63%)	6 (40%)	0.968
RT dose Hp cGy, median (IQR)	6000 (5300 - 6300)	6300 (5985 - 6700)	0.153
Adjuvant CT, n (%)	5 (15.15%)	7 (46.67%)	0.031*
Frozen number, median (IQR)	5 (4 - 5)	5 (4 - 5)	0.477
Pathologic differentiation, n (%)			0.497
wd	1 (3.57%)	0 (0%)	
md	12 (42.86%)	5 (41.67%)	
md to pd	8 (28.57%)	6 (50%)	
pd	7 (25%)	1 (8.33%)	
Hp Permanent specimen margin, n (%)			1
Negative	23 (71.88%)	11 (73.33%)	
Positive	9 (28.13%)	4 (26.67%)	
Margin distance (mm), median (IQR)	1 (1 - 2.25)	1 (1 - 1.25)	0.391
Lymphovascular invasion, n (%)	7 (21.21%)	3 (20%)	1
Node ENE, n (%)	2 (6.25%)	6 (40%)	0.009**
NG, n (%)	5 (15.2%)	1 (6.67%)	0.409
Organ preserved, n (%)	33 (100%)	14 (93.33%)	0.313
2nd ca., n (%)	16 (48.48%)	7 (46.67%)	0.907

Mann-Whitney U test. Chi-Square test. Fisher’s exact test. Fisher-Freeman-Halton Exact test. *p<0.05, **p<0.01

Eight patients (16.7%) developed a local recurrence in the hypopharynx; 6 of these patients underwent salvage transoral minimally invasive surgery—TORS (*n* = 4) or transoral laser microsurgery (*n* = 2)—and remained free of hypopharyngeal disease thereafter. Only 2 patients had persistent cancer of the hypopharynx at the time of death. Among the 15 deaths, 8 patients died with one or more distant metastases (lungs [*n* = 3], liver [*n* = 2], mediastinum [*n* = 2], bone [*n* = 1], brain [*n* = 1]), making distant spread the leading cause of death. Five patients died of second primary cancers (esophagus [*n* = 3], stomach [*n* = 1], adenocarcinoma of the lung [*n* = 2]).

**Table 4:** Comparison of data between disease recurrence group and no recurrence group

Recurrence	No (*n *= 34)	Yes (*n *= 14)	*p* value
Age, median (IQR)	57.58 (52.61 - 64.45)	53.94 (50.21 - 61.18)	0.441
Male, n (%)	33 (97.06%)	14 (100%)	1
Smoke/Alchoc/Betal, n (%)	32 (94.12%)	11 (78.57%)	0.14
Smoke, n (%)	29 (85.29%)	11 (78.57%)	0.676
Alcohol, n (%)	27 (79.41%)	10 (71.43%)	0.708
Betal, n(%)	17 (50%)	6 (42.86%)	0.653
1 st ca. in life, n (%)	32 (94.12%)	14 (100%)	1
Hx of other cacner, n (%)	2 (5.88%)	0 (0%)	1
Diagnosis site, n (%)			1
In hospital	25 (73.53%)	11 (78.57%)	
Outside hosptial	9 (26.47%)	3 (21.43%)	
Clinical T stage, n (%)			0.65
T1	12 (35.29%)	6 (42.86%)	
T2	17 (50%)	5 (35.71%)	
T3	5 (14.71%)	3 (21.43%)	
Clinical N stage, n (%)			0.618
N0	19 (55.88%)	7 (50%)	
N1	8 (23.53%)	2 (14.29%)	
N2	6 (17.65%)	4 (28.57%)	
N3	1 (2.94%)	1 (7.14%)	
Clinical overall stage, n (%)			0.66
I	8 (23.53%)	4 (28.57%)	
II	9 (26.47%)	3 (21.43%)	
III	10 (29.41%)	2 (14.29%)	
IVA	6 (17.65%)	4 (28.57%)	
IVB	1 (2.94%)	1 (7.14%)	
Clinical overall stage, n (%)			0.716
I	8 (23.53%)	4 (28.57%)	
II	9 (26.47%)	3 (21.43%)	
III	10 (29.41%)	2 (14.29%)	
IV	7 (20.59%)	5 (35.71%)	
Pathology T stage, n (%)			0.615
T0	0 (0%)	1 (7.69%)	
T1	18 (64.29%)	8 (61.54%)	
T2	8 (28.57%)	3 (23.08%)	
T3	2 (7.14%)	1 (7.69%)	
Pathology N stage, n (%)			0.014*
N0	18 (54.55%)	7 (50%)	
N1	9 (27.27%)	2 (14.29%)	
N2	5 (15.15%)	0 (0%)	
N3	1 (3.03%)	5 (35.71%)	
Pathology overall stage, n (%)			0.032*
I	10 (35.71%)	5 (38.46%)	
II	3 (10.71%)	1 (7.69%)	
III	9 (32.14%)	2 (15.38%)	
IVA	5 (17.86%)	0 (0%)	
IVB	1 (3.57%)	5 (38.46%)	
Pathology overall stage, n (%)			0.032*
I	10 (35.71%)	5 (38.46%)	
II	3 (10.71%)	1 (7.69%)	
III	9 (32.14%)	2 (15.38%)	
IVA	5 (17.86%)	0 (0%)	
IVB	1 (3.57%)	5 (38.46%)	
Pathology overall stage, n (%)			0.638
I	10 (35.71%)	5 (38.46%)	
II	3 (10.71%)	1 (7.69%)	
III	9 (32.14%)	2 (15.38%)	
IV	6 (21.43%)	5 (38.46%)	
Sites, n (%)			0.582
pyriform sinus	29 (85.29%)	11 (78.57%)	
post pharyngeal wall	4 (11.76%)	3 (21.43%)	
pyriform sinus+post cricoid mucosa	1 (2.94%)	0 (0%)	
側性, n (%)			0.562
R	18 (52.94%)	6 (42.86%)	
L	14 (41.18%)	8 (57.14%)	
RL	2 (5.88%)	0 (0%)	
Induction CT, n (%)	12 (35.29%)	4 (28.57%)	0.746
Tracheostomy, n (%)	2 (5.88%)	3 (21.43%)	0.14
Neck dissection, n (%)			0.654
ipsilateral	32 (94.12%)	13 (92.86%)	
bilateral	1 (2.94%)	1 (7.14%)	
Adjuvant RT, n (%)			0.169
no	18 (52.94%)	5 (35.71%)	
neck	2 (5.88%)	4 (28.57%)	
hp	2 (5.88%)	0 (0%)	
neck+hp	12 (35.29%)	5 (35.71%)	
RT dose neck cGy, n (%)	14 (41.18%)	9 (64.29%)	0.145
RT dose neck cGy, median (IQR)	6000 (5040 - 6600)	6000 (5970 - 6600)	0.368
RT dose Hp cGy, n (%)	14 (41.18%)	5 (38.46%)	0.865
RT dose Hp cGy, median (IQR)	6000 (5450 - 6150)	6600 (5970 - 6600)	0.226
Adjuvant CT, n (%)	6 (17.65%)	6 (42.86%)	0.139
Frozen number, median (IQR)	5 (4 - 5)	5 (5 - 6)	0.021*
Pathologic differentiation, n (%)			0.402
wd	1 (3.7%)	0 (0%)	
md	9 (33.33%)	8 (61.54%)	
md to pd	11 (40.74%)	3 (23.08%)	
pd	6 (22.22%)	2 (15.38%)	
Hp Permanent specimen margin, n (%)			1
Negative	25 (73.53%)	9 (69.23%)	
Positive	9 (26.47%)	4 (30.77%)	
Margin distance (mm), median (IQR)	1 (1 - 2)	1 (1 - 1.75)	0.392
Lymphovascular invasion, n (%)	8 (23.53%)	2 (14.29%)	0.701
Node ENE, n (%)	3 (8.82%)	5 (38.46%)	0.028*
NG, n (%)	3 (8.82%)	4 (28.57%)	0.171
Organ preserved, n (%)	34 (100%)	13 (92.86%)	0.292
2nd ca., n (%)	19 (55.88%)	4 (28.57%)	0.085

Chi-Square test. Fisher’s exact test. Fisher-Freeman-Halton Exact test. *p<0.05, **p<0.01

**Fig. 1:**
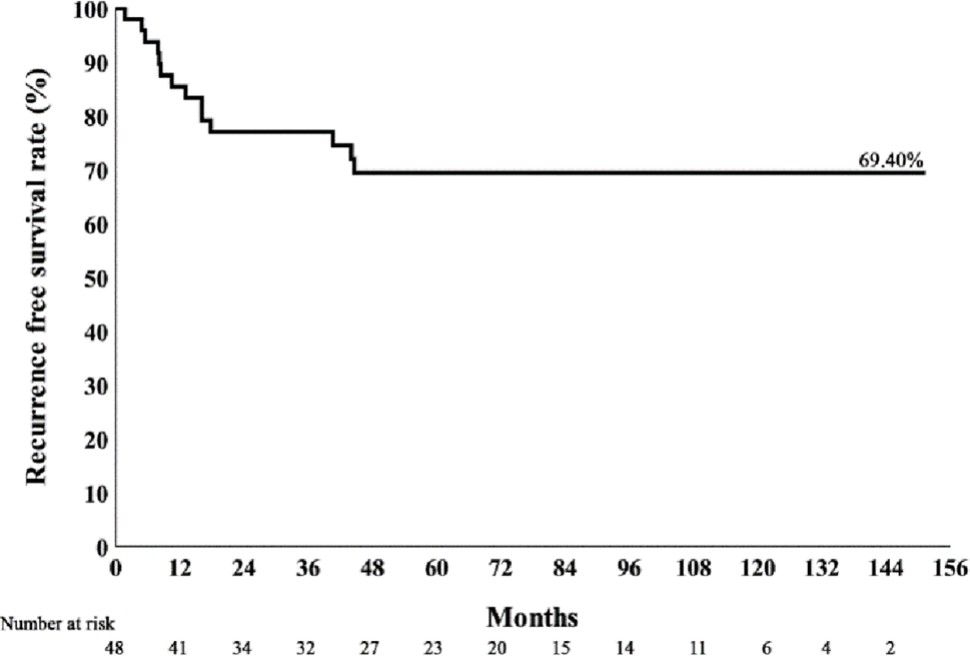
Kaplan–Meier curve of recurrence-free survival over 10 years

**Fig. 2:**
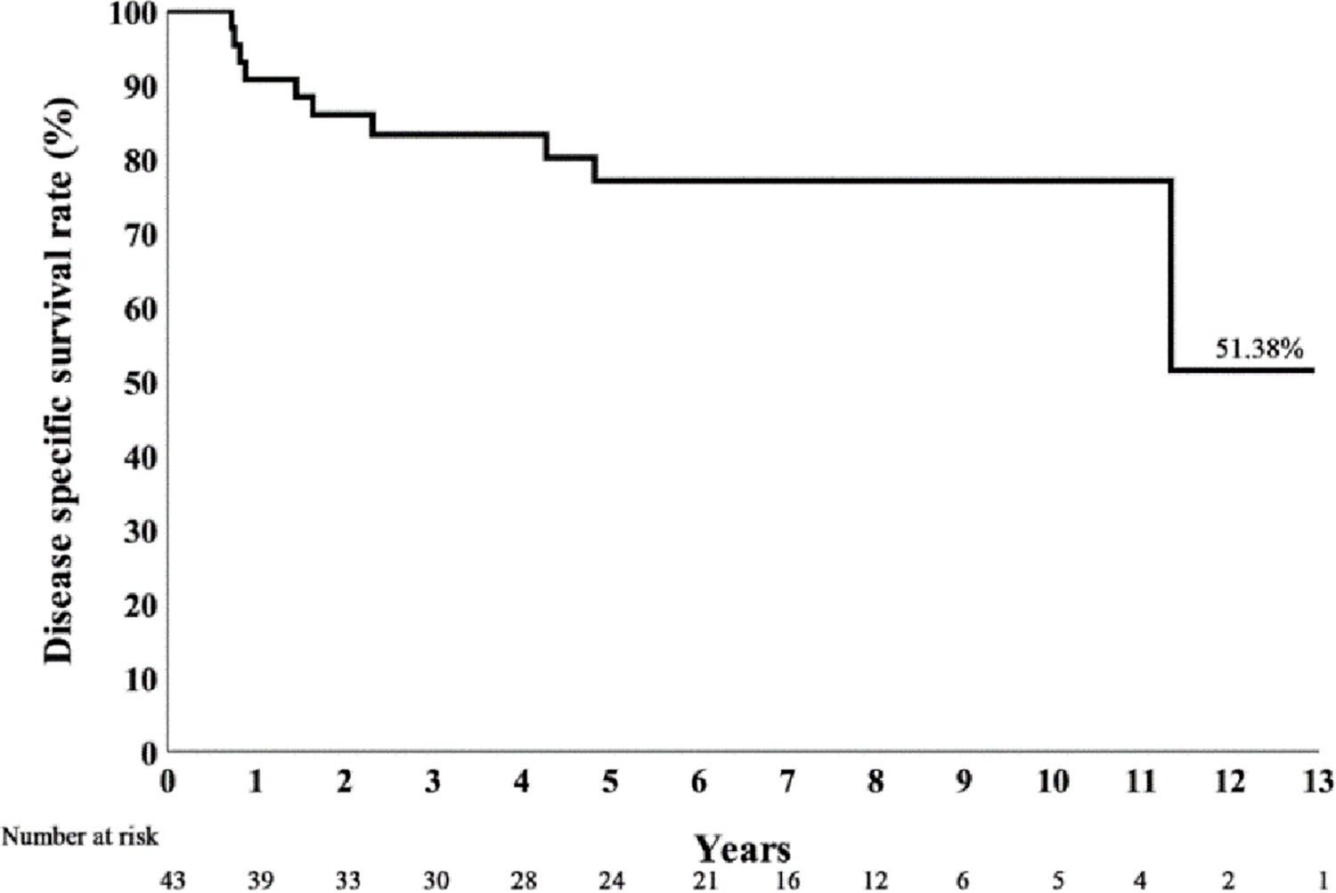
Kaplan–Meier curve of disease-specific survival over 10 years

**Fig. 3:**
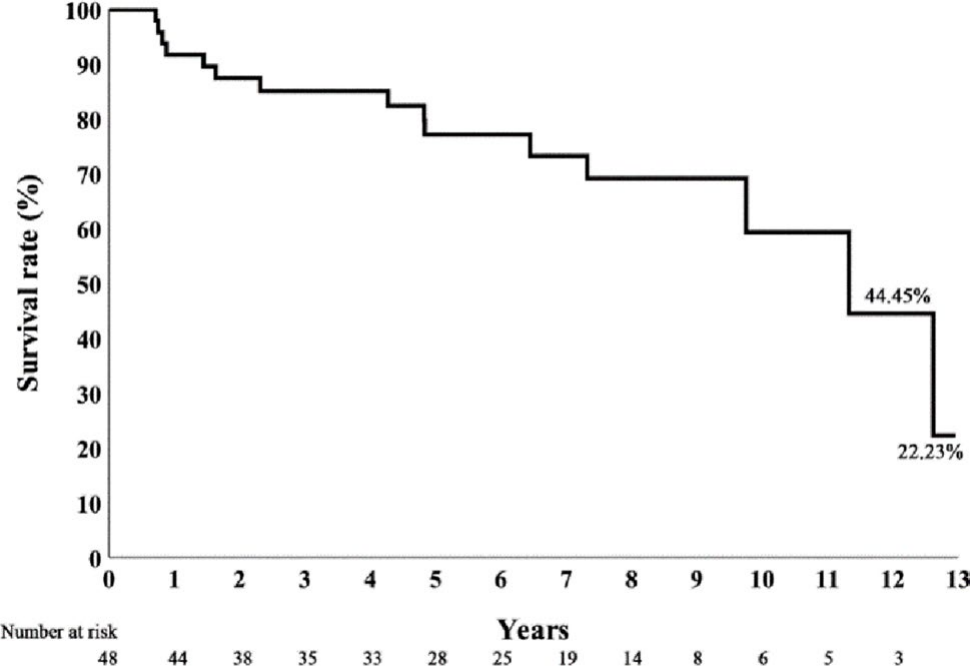
Kaplan–Meier curve of overall survival over 10 years

**Fig. 4:**
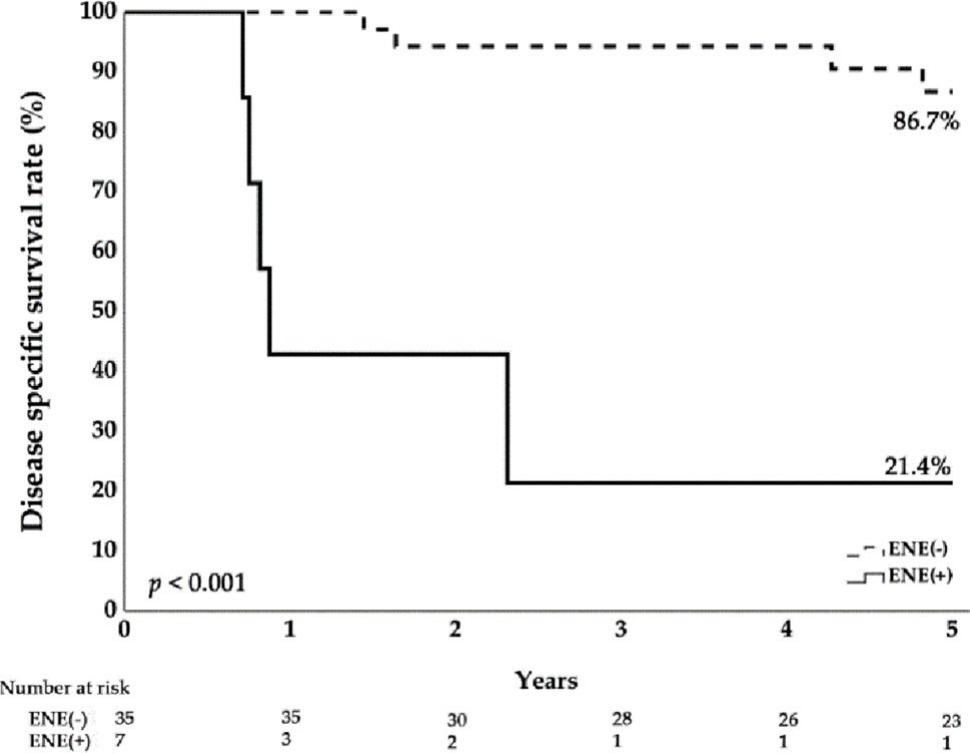
Extra-nodal extension significantly reduces the disease specific survival rate

Kaplan–Meier curves for 10-year recurrence-free survival (RFS), disease-specific survival (DSS), and overall survival (OS) are shown in Figs. 1, 2 and 3. ENE significantly reduces the disease specific survival rate (Fig. 4). The 5-year RFS was 69%, and DSS was 77% at both 5 and 10 years. Organ preservation was maintained in 100% of survivors and in 93.3% of patients in the mortality group until death. Six patients (12.5%) required nasogastric tube feeding at the last follow-up. Over long-term follow-up, 23 patients (47.9%) developed at least one second primary cancer. At the last follow-up, five surviving patients with nasogastric tubes had lived for 2 to 11 years after surgery. One patient, during his 3-year follow-up, was diagnosed with Sjögren’s syndrome and a second primary tonsil cancer, which was complicated by a retropharyngeal abscess after treatment. Another patient developed two additional cancers (floor of mouth and oropharynx) during his 5-year follow-up; he underwent three surgeries and received two courses of radiation therapy. A third patient had two additional cancers during his 11-year follow-up (contralateral oropharynx and ipsilateral tongue base), for which he received a total of three surgeries and two courses of radiation therapy. The remaining two patients had advanced stage III–IV cancers and received more than 60 Gy of adjuvant radiation after transoral robotic surgery (TORS). High-dose adjuvant radiation and multiple primary cancers in the upper aerodigestive tract significantly compromised their swallowing function.

**Table 5:** Univariate and multivariable Cox/logistic regression analysis of prognostic factors for patient survival

	Cox				Logistic			
	Simple model		Multiple model		Simple model		Multiple model	
	HR (95%CI)	*p* value	HR (95%CI)	*p* value	OR (95%CI)	*p* value	OR (95%CI)	*p* value
Clinical N stage								
N0	1.00				1.00			
N1	1.01 (3.80 - 14.31)	0.049*			0.87 (4.20 - 20.34)	0.075		
N2	0.90 (3.85 - 16.41)	0.068			0.57 (2.80 - 13.83)	0.206		
N3	1.43 (16.27 - 185.17)	0.025*			0.22 (4.20 - 79.32)	0.338		
Pathology N stage								
N0	1.00				1.00			
N1	---				---			
N2	0.07 (0.57 - 4.66)	0.601			0.05 (0.53 - 5.55)	0.597		
N3	5.35 (30.55 - 174.55)	<0.001**			1.06 (10.63 - 106.57)	0.045*		
Pathology overall stage								
I	1.00				1.00			
II	0.24 (1.32 - 7.31)	0.747			0.21 (2.00 - 18.69)	0.543		
III	---	---			---	---		
IVA	0.06 (0.57 - 5.08)	0.610			0.04 (0.50 - 5.74)	0.578		
IVB	4.67 (46.54 - 464.33)	0.001**			0.91 (10.00 - 110.28)	0.060		
Adjuvant RT								
no	1.00				1.00			
neck	1.77 (8.26 - 38.57)	0.007**			1.01 (7.20 - 51.39)	0.049*		
hp	0.26 (2.57 - 24.96)	0.417			0.19 (3.60 - 68.34)	0.394		
neck+hp	0.74 (3.14 - 13.36)	0.122			0.36 (1.50 - 6.32)	0.581		
Adjuvant CT	1.78 (5.35 - 16.04)	0.003**	0.73 (2.64 - 9.56)	0.140	1.22 (4.90 - 19.69)	0.025*	0.28 (1.78 - 11.33)	0.542
Node ENE	3.8 (12.93 - 44.03)	<0.001**	2.1 (8.44 - 33.83)	0.003*	1.71 (10.00 - 58.43)	0.011*	0.75 (6.62 - 58.61)	0.089

Cox/Logistic regression. *p<0.05, **p<0.01

**Table 6:** Univariate and multivariable Cox/logistic regression analysis of prognostic factors for patient recurrence

	Cox				Logistic			
	Simple model		Multiple model		Simple model		Multiple model	
	HR (95%CI)	*p* value	HR (95%CI)	*p* value	OR (95%CI)	*p* value	OR (95%CI)	*p* value
Clinical N stage								
N0	1.00				1.00			
N1	0.76 (0.16 - 3.68)	0.735			0.68 (0.11 - 4.01)	0.669		
N2	1.60 (0.47 - 5.49)	0.451			1.81 (0.39 - 8.39)	0.448		
N3	3.97 (0.47 - 33.5)	0.204			2.71 (0.15 - 49.53)	0.500		
Pathology N stage								
N0	1.00				1.00			
N1	0.65 (0.14 - 3.15)	0.596			0.57 (0.10 - 3.33)	0.534		
N2	---	---			---	---		
N3	8.66 (2.44 - 30.75)	0.001**			12.86 (1.27 - 130.54)	0.031*		
Pathology overall stage								
I	1.00				1.00			
II	0.55 (0.06 - 4.71)	0.582			0.67 (0.05 - 8.16)	0.751		
III	0.48 (0.09 - 2.49)	0.384			0.44 (0.07 - 2.89)	0.396		
IVA	---	---			---	---		
IVB	7 (1.78 - 27.44)	0.005**			10.00 (0.91 - 110.28)	0.060		
Adjuvant RT								
no	1.00				1.00			
neck	4.56 (1.22 - 17.06)	0.024*			7.20 (1.01 - 51.39)	0.049*		
hp	---	---			---	---		
neck+hp	1.46 (0.42 - 5.04)	0.550			1.50 (0.36 - 6.32)	0.581		
Adjuvant CT	2.97 (1.03 - 8.60)	0.045*			3.50 (0.88 - 13.88)	0.075		
Node ENE	4.93 (1.59 - 15.29)	0.006**	4.36 (1.37 - 13.88)	0.013*	6.46 (1.27 - 32.92)	0.025*	5.91 (1.13 - 30.89)	0.035*
NG	3.57 (1.10 - 11.64)	0.035*	2.51 (0.65 - 9.74)	0.183	4.13 (0.79 - 21.69)	0.093	2.49 (0.38 - 16.52)	0.345

Cox/Logistic regression. *p<0.05, **p<0.01

## Discussion

Hypopharyngeal cancer is uncommon, accounting for less than 5% of all head and neck malignancies in the global epidemiology study [14]. According to “The natural history of patients with squamous cell carcinoma of the hypopharynx,” Hall et al. [16] also noted that most oncologists have limited experience managing this disease. Furthermore, there is no level I evidence defining the optimal treatment, nor consensus on management [17–19]. In Hall’s retrospective, population-based review of 595 patients, although 279 (47%) had T1–T2 primary tumors, most presented with advanced disease (over 50% stage IV) due to lymph node metastasis. After curative therapy (surgery ± postoperative RT, or RT ± surgery for residual disease), 20% had residual disease; recurrences typically occurred within the first year, and 50% of first recurrences involved distant metastases. Overall, 47% were disease-free at 3 years, yet ultimately 64% died of their cancer. Consistently, the global survey by Mousavi SE et al. [14] reported mortality-to-incidence ratios as high as 0.45. Therefore, it is imperative to develop treatment strategies that improve survival without sacrificing organ structure or function. One option is the previously described nonsurgical chemoradiation (CRT) approach proposed by Lefebvre et al. [4]; however, CRT can cause severe toxicities, and preservation of anatomy does not necessarily equate to preservation of function—the combined use of chemotherapy and radiotherapy increases toxicity and adversely affects swallowing [20, 21]. Since Steiner and Herbst introduced transoral laser microsurgery (TLM) for hypopharyngeal cancer with favorable functional outcomes [22], minimally invasive transoral surgery combined with neck dissection, followed by pathology-guided adjuvant radiotherapy, has become a feasible strategy.

### Outcomes of transoral surgery in the literature

Although the number of reports is limited, outcomes of transoral surgery for hypopharyngeal cancer have been described in the literature. Rane C et al. published a systematic review and meta-analysis of 6 studies (2013–2018) on TLM in 2020 [23–29]. Most patients had early (T1–T2) primary cancers, with a cumulative proportion of 64% (range, 38%–100%). Cancer subsites were predominantly the pyriform sinus (82%), followed by the posterior pharyngeal wall (11%), and less commonly the postcricoid region (6.3%). The neck-dissection rate was 84% (range, 70%–100%), and the postoperative radiotherapy rate was 71% (range, 43%–86%). In our series, most patients also had early T1–T2 hypopharyngeal cancer with a similar subsite distribution. With a 97.9% neck-dissection rate, the proportion receiving postoperative irradiation to the digestive tract was reduced to ~ 40%, with a median dose of 6000 cGy. Our 5-year overall survival of 77% exceeds the highest overall-survival rate of 59% reported by Hung et al. [29], and our 5-year disease-specific survival matches their result at 77%. Our 5-year recurrence-free survival was 69%, which falls between the 82% reported in Rudert’s 29-patient cohort (mostly early T stage) [24] and the 55.9% reported in Weiss’s 211-patient cohort (mostly advanced T stage) [27].

With technological advances, several other transoral approaches have been developed, and transoral robotic surgery (TORS) has attracted increasing attention over the past decade. In Armando’s systematic review and meta-analysis [30], the cumulative overall survival was 85.5%, higher than the 58.5% observed at ≥ 36 months of follow-up. As a minimally invasive technique, TLM has limitations, including geometric constraints from the microscope’s line of sight; cancers are often removed piecemeal, making margin assessment more challenging. Although published reports remain limited, our previous studies [12, 13] and results from other institutions [10, 31–34] support the utility of TORS in hypopharyngeal cancer. To date, our cohort has the longest median follow-up (6.2 years). Our 5-year overall survival of 69% is similar to Mazerolle’s 66% [34] in early-stage hypopharyngeal cancer. As noted in prior studies, TORS offers several advantages for transoral surgery: a highly magnified panoramic view of the hypopharynx enabling en bloc cone-shaped excision of the pyriform sinus; EndoWrist instruments that permit precise, angled dissection with motion scaling and tremor reduction; and a “two-surgeon, four-hand” setup that facilitates smoke evacuation, soft-tissue manipulation, secure hemostasis via vessel ligation, and improved exposure of the narrow hypopharynx through traction and counter-traction [12, 13].

### Neoadjuvant chemotherapy

**Fig. 5:**
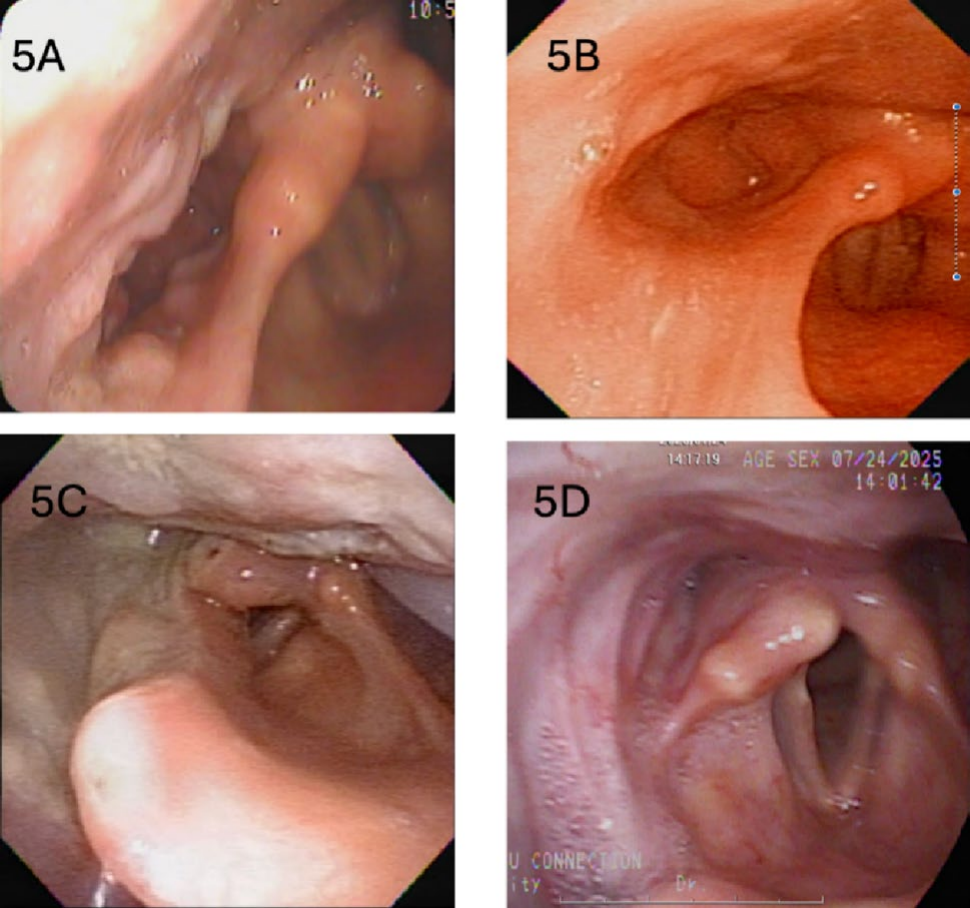
Flexible laryngoscopy in a patient with hypopharyngeal cancer. **A**, Baseline T3 cancer of the right pyriform sinus. **B**, Marked reduction in tumor volume after cisplatin-based neoadjuvant chemotherapy. **C**, Post-TORS mucosal healing. **D**, Nine-year follow-up after de-escalated adjuvant radiotherapy (6000 cGy) shows no local recurrence with normal laryngeal and swallowing function

In our study, 12 (35.3%) patients received cisplatin-based chemotherapy prior to TORS, either because of T3 tumors or because limited robotic-surgery slots delayed TORS. In the literature, NACT before surgery has been evaluated mainly in oral cavity cancers. Although Licitra L et al. [35] reported that adding primary chemotherapy to standard surgery did not improve survival, in their study primary chemotherapy appeared to reduce the number of patients requiring mandibulectomy and/or radiotherapy. Similarly, our previous report [13] and the study by Park YM et al. [36] suggest that NACT can reduce tumor volume in the narrow hypopharynx and facilitate TORS using EndoWrist instruments. In the NACT and TORS for Human Papillomavirus–related oropharyngeal cancer (NECTORS) trial [37], Sadeghi N et al. also concluded that this strategy is an effective treatment option for patients with stage III and IVa HPV-OPSCC. However, neoadjuvant chemotherapy was only given on an ad-hoc basis rather than under any protocol. Although our data indicate that NACT before TORS is a feasible approach, careful interpretation of our results and further investigation were warranted. (Fig. 5)

### Neck dissection

In our cohort, concordance between clinical and pathologic N category was only 60.4%. This has practical implications: nonsurgical chemoradiation paradigms may undertreat some patients (with consequent poorer survival) or overtreat others (with greater complications). In particular, the pathologic N category—and the resultant overall pathologic stage—were significantly associated with survival in our study. The prevalence of extranodal extension (ENE) was also markedly higher in the mortality group (40.0%) than in the survivor group (6.3%). Specifically, in the Cox regression and logistic regression models (Tables 5 and 6), patients with ENE exhibited significantly higher hazard ratios or odds ratios for mortality and disease recurrence. In AJCC staging for head and neck cancers, extranodal extension (ENE) was incorporated into nodal staging in the 8th edition for most sites, including the hypopharynx [38]. When present on pathologic examination, ENE upstages the pN category by one level (e.g., pN1→pN2; ENE + large/bulky disease may be pN3b). A report from Taiwan likewise showed that 41% of 200 patients who had hypopharyngeal cancer(*N* = 82) were upstaged under AJCC 8th edition, entirely due to redistribution of the N category; the authors concluded that AJCC 8th improves survival prediction in hypopharyngeal cancer compared with the 7th, particularly owing to inclusion of ENE status and revisions to nodal classification [39]. These findings further emphasize the importance of neck dissection to determine the true nodal stage and to plan adjuvant therapy precisely.

### Cut margins at hypopharynx

Obtaining tumor-free cut margins is essential in reducing the risk of local recurrences. However, there is no universal definition of what constitutes a clear or close margin. In the literature, the optimal cutoff for a safe margin in oral cancer varies widely, from 1 mm [40, 41] to more than 4 mm [42]. In recent publications on TORS for oropharyngeal cancer, the definition of a close surgical margin is likewise nonstandard, ranging from < 1 to 5 mm [43]. Compared with the oral cavity, the hypopharynx is relatively small; in the study by Aminpour S et al., the mean thickness of the pharyngeal wall at rest was 0.39 cm (± 0.09) in younger adults and 0.30 cm (± 0.08) in older adults [44]. Accordingly, achieving very wide margins via transoral surgery for hypopharyngeal cancer is unrealistic. Nonetheless, with highly magnified 3-D endoscopy, we can meticulously inspect the mucosa to secure a clear cut margin. In addition, we submitted multiple frozen-section specimens to prevent the presence of microscopic carcinoma at the margins. In our cohort, the local recurrence rate was 16.7% at a median follow-up of 6.2 years. Margin status (positive vs. negative) on the en-bloc specimen was not associated with survival or local recurrence in either univariate or multivariable analysis. Theoretically, the lack of tactile feedback in robotic surgery limits the surgeon’s ability to prevent specimen tear on the thin and fragile hypopharyngeal mucosa, which may lead to false-positive margin status on the permanent specimen. However, our rigorous protocol of combining multiple intraoperative frozen sections with tailored postoperative therapy effectively “sterilized” the surgical bed, thereby decoupling margin status from local failure or mortality.

### Cause of death in long term follow up

In our study, after 5-years, the local recurrence free survival and disease specific survival remained unchanged. In patients with mortality, mostly caused by distant organ metastasis. In Japan, Tateda et al. [45] also have the same findings. Even if locoregional control is accomplished, distant metastasis or multiple primary cancers emerged in the long-term follow-up, resulting in a poor prognosis.

In addition to distant metastasis, 23 (47.92%) of patients in our study had at least one second primary cancer at other upper aerodigestive mucosae, or other sites of the body. For other upper aerodigestive cancers, active surveillance and early detection may successfully control the disease. However, 3 patients died of esophageal cancer, 1 died of stomach cancer, and 1 died of lung adenocarcinoma. Another population-based study from Taiwan also emphasizes that Routine Endoscopy for Esophageal Cancer Is Suggestive for Patients with Oral, Oropharyngeal and Hypopharyngeal Cancer [46]. Chung CS et al. [47] also suggested that screening and surveillance of esophageal second primary cancer by magnifying endoscopy with narrow band imaging improves the survival of patients with hypopharyngeal cancer.

### Study limitations

**Fig. 6:**
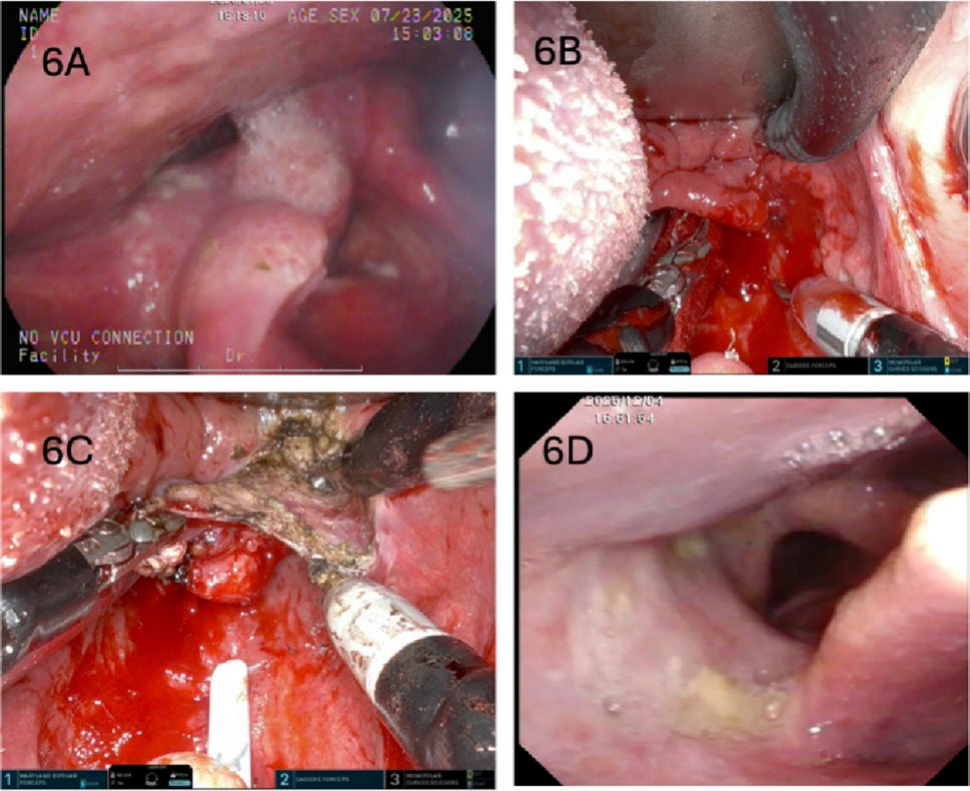
Single-port TORS for a hypopharyngeal cancer initially read as carcinoma in situ on two separate transoral laser incisional biopsies. **A**, Initial view of a right pyriform sinus cancer involving the aryepiglottic fold. **B**, Exposure with 3 single-port da Vinci instruments placed in the hypopharynx (Maryland bipolar forceps, Cadiere forceps, and monopolar scissors). **C**, Three-instrument setup allows improved traction and countertraction. **D**, En-bloc excisional biopsy via TORS confirmed invasive squamous cell carcinoma; 3-month postoperative view shows a healed wound with normal vocal fold mobility

This study has several limitations. First, it is a retrospective, single-center investigation conducted over a long accrual period. Given the rarity of hypopharyngeal cancer and the paucity of long-term TORS reports, our findings should be interpreted cautiously, although they illustrate the potential of combining robotic transoral resection with neck dissection to establish the true pathologic stage and to tailor adjuvant therapy. Second, the study lacks a control arm; Although indirect comparisons with existing meta-analyses show our results align with previously reported ranges, cross-study comparisons have inherent limitations. In future studies, we plan to compare this management with non-surgical organ preservation approaches to better evaluate its value. Third, the present study is limited by a relatively small sample size and a low number of outcome events. Under these conditions, performing a fully adjusted multivariable survival analysis (e.g., Cox regression) would risk model overfitting and unstable estimates. To strengthen the statistical analysis within these constraints, we conducted additional Logistic regression analyses as sensitivity analyses to explore the robustness of the observed associations. The results of these regression analyses were consistent with the primary univariate findings, supporting the stability of the observed associations despite the limited adjustment capacity. Fourth, patient selection remains a critical factor; local tumor factors, such as significant extension to the post-cricoid mucosa, involvement of cartilage, or extension through the thyrohyoid membrane, remain contraindications for TORS in our practice. Furthermore, patients with severe pre-existing dysphagia requiring tube feeding may not be ideal candidates for this organ-preservation strategy. Fifth the current dimensions of the da Vinci EndoWrist instruments are not optimized for transoral work in the hypopharynx. Although our team is experienced with TORS in this narrow space, the technique has not yet been widely disseminated. Future device iterations—including the single-port (SP) system now available at our institution, which permits three working instruments for improved traction and counter-traction—may enhance feasibility and adoption (Fig. 6). Continued development of ENT-specific instruments is warranted.

## Conclusion

TORS is a feasible option for treating T1–T2 and selected T3 hypopharyngeal cancers without distant metastasis. When combined with neck dissection, it enables accurate pathologic staging to guide adjuvant therapy. Despite durable locoregional control, distant organ metastasis remains the leading cause of death.

## Data Availability

No datasets were generated or analysed during the current study.
